# Socioeconomic differences in COVID-19 infection, hospitalisation and mortality in urban areas in a region in the South of Europe

**DOI:** 10.1186/s12889-022-14774-6

**Published:** 2022-12-12

**Authors:** Nicolás F Fernández-Martínez, Rafael Ruiz-Montero, Diana Gómez-Barroso, Alejandro Rodríguez-Torronteras, Nicola Lorusso, Inmaculada Salcedo-Leal, Luis Sordo

**Affiliations:** 1 Unidad de Gestión Clínica Medicina Preventiva y Salud Pública, Hospital Universitario Reina Sofía, Córdoba, 14004 Spain; 2 Instituto Maimónides de Investigación Biomédica de Córdoba (IMIBIC), Córdoba, Spain; 3 Centro Nacional de Epidemiología, Instituto de Salud Carlos III, Madrid, Spain; 4 CIBER en Epidemiología y Salud Pública (CIBERESP), Barcelona, Spain; 5 Dirección General de Salud Pública, Consejería de Salud y Consumo, Junta de Andalucía, Spain; 6 Departamento de Salud Pública y Materno-Infantil, Facultad de Medicina, Universidad Complutense de Madrid, Madrid, Spain

**Keywords:** COVID-19, Urbanization, Geographical, Mortality, Hospitalisation, Socio-economic status

## Abstract

**Background:**

To analyse differences in confirmed cases, hospitalisations and deaths due to COVID-19 related to census section socioeconomic variables.

**Methods:**

Ecological study in the 12 largest municipalities in Andalusia (Spain) during the first three epidemic waves of the COVID-19 (02/26/20—03/31/21), covering 2,246 census sections (unit of analysis) and 3,027,000 inhabitants. Incidence was calculated, standardised by age and sex, for infection, hospitalisation and deaths based on average gross income per household (AGI) for the census tracts in each urban area. Association studied using a Poisson Bayesian regression model with random effects for spatial smoothing.

**Results:**

There were 140,743 cases of COVID-19, of which 12,585 were hospitalised and 2,255 died. 95.2% of cases were attributed to the second and third waves, which were jointly analysed. We observed a protective effect of income for infection in 3/12 cities. Almeria had the largest protective effect (smoothed relative risk (SRR) = 0.84 (0.75–0.94 CI 95%). This relationship reappeared with greater magnitude in 10/12 cities for hospitalisation, lowest risk in Algeciras SRR = 0.41 (0.29–0.56). The pattern was repeated for deaths in all urban areas and reached statistical significance in 8 cities. Lowest risk in Dos Hermanas SRR = 0.35 (0.15–0.81).

**Conclusions:**

Income inequalities by geographical area were found in the incidence of COVID-19. The strengths of the association increased when analysing the severe outcomes of hospitalisations and, above all, deaths.

**Supplementary Information:**

The online version contains supplementary material available at 10.1186/s12889-022-14774-6.

## Background

The coronavirus disease of 2019 (COVID-19) has been marked by the lack of prior knowledge of its characteristics, an initial underestimation of its impact and delayed decision-making in terms of healthcare measures and public health [1]. This has resulted in very different disease management in different countries, according to the profiles of their inhabitants [2].

Despite these differences, evidence suggests that COVID-19 has had a greater effect on populations with worse socioeconomic characteristics [3, 4], and it has been particularly acute, as with other infectious diseases, in urban populations [5, 6]. In urban areas, contact between people is more frequent than in rural areas, especially in areas with worse social determinants of COVID-19, such as working and living conditions [7].

Throughout the pandemic, the consequences of these socioeconomic inequalities in the urban context have not been limited to infections. There have also been higher mortality rates [8] and hospitalisation rates [9] in underprivileged urban areas. It should be noted that many studies were conducted at different time points early in the pandemic or were carried out without differentiating between different time points, which might contribute to the inconsistent findings [10, 11]. Hospitalisations and mortality due to COVID-19 are the two issues that have most affected political and public health measures, such as confinements and curfews, and have put the health care infrastructure at risk [11, 12].

In many countries, including Spain, COVID-19 has had an impact during two different time periods with very different characteristics [10]. There are notable differences between the first wave, which began in February of 2020, in which there was scant awareness of the disease compared to the following waves. The first wave in Spain provoked greater excess mortality than in any other European country [13]. However, this wave hit certain zones hard, while in others it was much less relevant. In Andalusia, for example, confinement took place when there were still very few cases in the region [14]. By mid-July, when new infections started to rise again (with the beginning of the second wave), more than 27,000 deaths had been officially registered in Spain as cases of COVID-19 [14, 15]. Only five per cent came from Andalusia, a region with 17.9 per cent of the Spanish population. COVID-19 had a much greater impact in Andalusia during the second and third waves, accounting for around 30 per cent of cases and deaths in Spain [14].

Despite the existence of research on the association between sociodemographic risk factors and COVID-19, especially concerning urban areas [3, 4, 7, 16, 17], there are few studies that evaluate the risk of infection, hospitalisation and death due to the virus in a single study population. Furthermore, in order to accurately interpret the influence of socioeconomic factors on these outcomes, biased data from the first wave (diagnostic bias) should be considered separately [11].

The objective of this study was to analyse socioeconomic differences in the 12 primary urban areas in Andalusia (Spain) in terms of confirmed cases, hospitalisations and deaths due to COVID-19 during the first three epidemic waves of the pandemic.

## Materials and methods

We carried out an ecological, retrospective study of the first three epidemic waves of the COVID-19 pandemic in the primary urban areas in Andalusia, Spain.

### Variables

The units of analysis were the census sections (small territorial units inside a neighbourhood encompassing approximately between 1,000 and 2,500 inhabitants) in urban areas (territorial units defined by population volume criteria and by territorial features). The sociodemographic variables were: the number of inhabitants (aggregated and disaggregated by sex and age) and average annual gross income per household (AGI) in euros. The epidemiological variables were: confirmed cases of COVID-19, as defined by the Spanish Ministry of Health (which mainly considered the laboratory criteria of confirmed PCR or antigen test to SARS-CoV-2, depending on the period of the pandemic [18]), hospitalisation of confirmed COVID-19 cases, deaths of confirmed cases of COVID-19, and epidemic waves, defined by the acceleration and deceleration of the number of new cases of COVID-19 (first epidemic wave: February 26 [first confirmed case in Andalusia] to July 12, 2020; second wave: July 13 to December 27, 2020; third wave: December 28, 2020 to March 31, 2021).

### Information sources

The most recent available information on demographic data (2019) and AGI (2018) came from the National Statistics Institute (NSI) [19]. However, given that AGI in 2018 was available for 99.1 per cent of census sections, information for prior years was used for the rest: 2017 (0.3%), 2016 (0.1%) and 2015 (0.4%). In 0.2 per cent of census sections there were no available data on AGI, thus they were excluded from the analysis. The confirmed COVID-19 cases in terms of diagnosis, hospitalisation and deaths—geocoding (X and Y coordinates)- and the information on institutionalisation in elderly or disability care centres (these cases were excluded due to the risk of interference with the spatial analysis) were obtained from the Epidemiological Surveillance System of Andalusia (SVEA). Finally, in order to establish cut-off points between epidemic waves, information on the evolution of the pandemic in the study area was obtained from the Andalusian Institute of Statistics and Cartography [20].

The dependent variable was calculated using the standardised incidence ratio (SIR) for COVID-19 for three outcomes of interest: cases of infection, hospitalisation and deaths. The independent variables were AGI, age and sex (these were used for the standardisation of rates).

### Study area

**Fig. 1:**
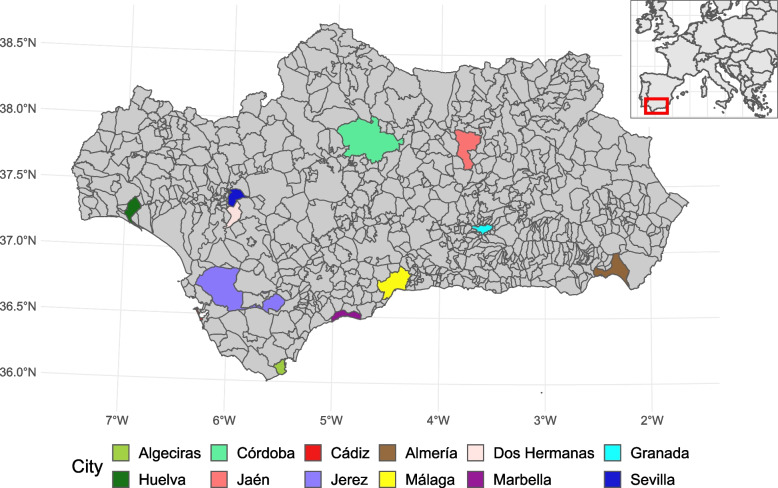
Location of studied urban areas

This study exclusively analysed the 12 municipalities in Andalusia with a population over 100,000 inhabitants [21]. In total, these urban areas bring together 2,246 census sections and 3,027,000 inhabitants, which represents 36 per cent of the population of Andalusia and 6 per cent of the total population in Spain (Fig. 1).

### Statistical analysis

The first wave was analysed individually, and the second and third waves were analysed jointly. The reason for dividing the period into two-time windows- and for our work, focusing on the second of these windows- is that the data related to the first wave were biased due to the limited accessibility of COVID-19 diagnostic tests [10, 13, 22]. In contrast, despite the existence of differences, the second and third waves are reasonably comparable due to uniformity in the definition of a confirmed case and the general availability of the SARS-CoV-1 antigen tests, thus they were analysed together. The temporal stratification was carried out in terms of the first wave vs the second and third waves. The spatial analysis using the Bayesian model was carried out stratifying by urban area, so each urban area was analysed independently. In summary, three outcomes were studied: the standardised incidence ratio (SIR) of COVID-19 at the case and hospitalisation levels and the standardised mortality ratio (SMR) of COVID-19, in 12 urban areas and in two time windows.

In order to carry out the statistical analysis, the SIR of COVID-19 was calculated, standardised by sex and age (categorised into 21 quinquennial groups). The SIR was obtained using the quotient of the observed and expected cases, by way of indirect standardisation, setting as reference (SIR = 1) the average ratio across census sections of all municipalities. Later, we employed a Bayesian spatial model to investigate spatial risk factors for the three aforementioned COVID-19 outcomes. In order to avoid the effect of small areas, we calculated the smoothed relative risk (SRR), setting as reference (SSR = 1) the average risk across census sections among each urban area. To do so, we used the model proposed by Besag, York and Mollié [23] of spatial smoothing using Poisson regression with random effects, which represents the heterogeneity of each geographic unit and the spatial contiguity, based on an auto-regressive conditional model (CAR).

$$0i \sim Po(Ei\;\lambda i)$$

$$log (\lambda i)=\alpha +hi+bi$$

where: *λi* is the relative risk in area i; *Oi* the number of cases in area i, α the constant, *Ei* the expected number of cases, *hi* the ordinary random-effects component for non-spatial heterogeneity among census sections and *bi* the spatial term.

Finally, the logarithm of AGI for each census section was added to the model as an independent covariable (analysed as a quantitative continuous variable). The statistical analysis was carried out with R software (version 4.0.3), using the spatial analysis package R-INLA.

### Patient and public involvement

Due to the ecological and non-interventional nature of the study we have not involved the public in the design or conduct of our research.

## Results

**Fig. 2:**
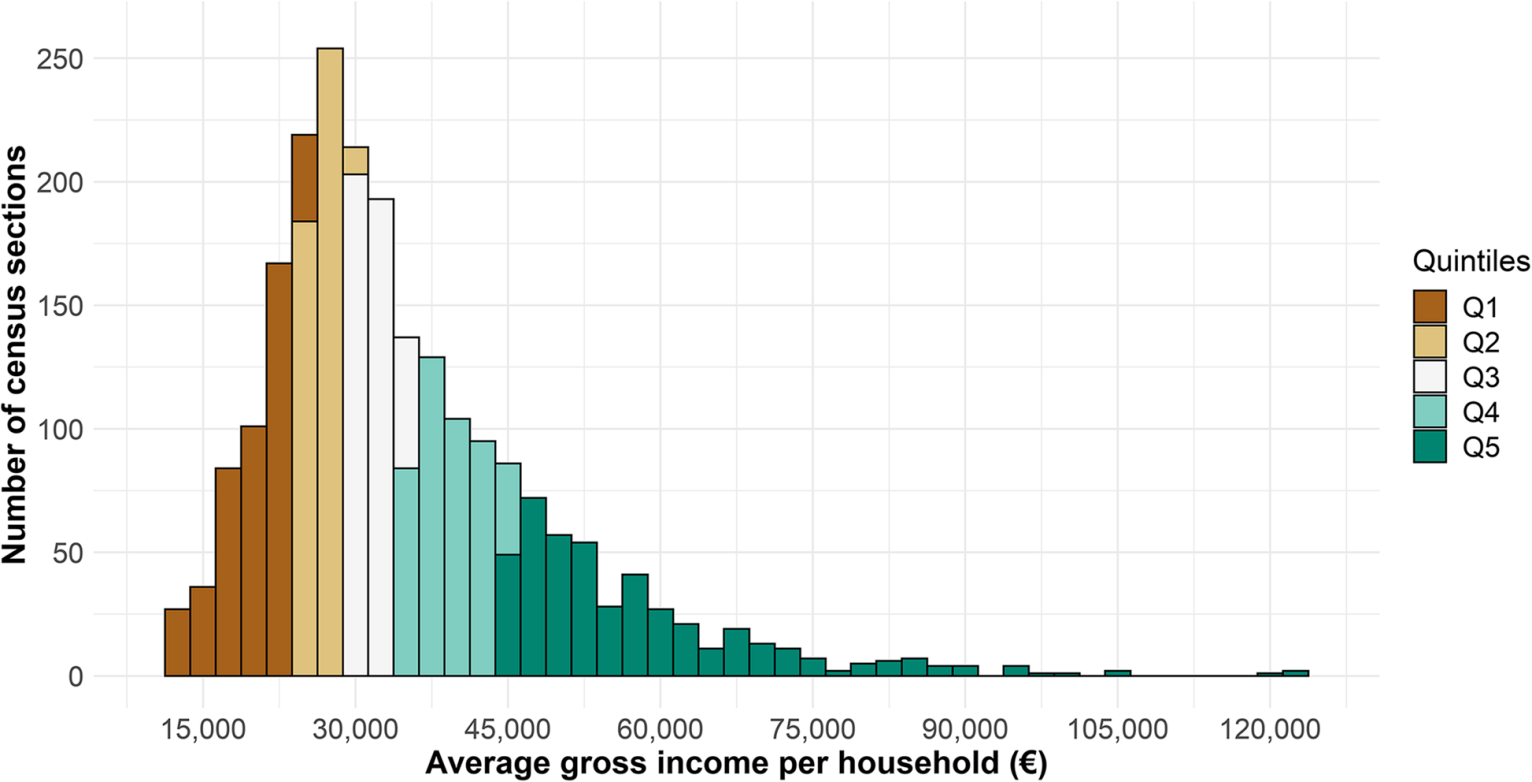
Distribution of average gross income by household in the studied urban areas, by income quintiles

Figure 1 shows the location of the urban areas studied. The distribution of AGI was non-normal and heterogeneous in all of them. The AGI ranged from 13,000€ in the census section with the lowest income to 123,000€ in the census section with the highest income, although both of the first two quintiles were under 30,000€ and the third was below 35,000€ (Fig. 2). This asymmetric distribution was similar among the different urban areas, though a greater dispersion was observed in the municipalities with larger populations (Sevilla and less so Málaga and Córdoba) (Supplementary File 1).

After excluding 3,952 institutionalised cases (2.4%), 16,547 cases without available geocoding (10.1%) and 1,859 cases geocoded outside the municipalities studied (1.1%), the number of confirmed cases of COVID-19 analysed was 140,743 (86.3% of the total confirmed cases reported to the SVEA), of which 12,585 were hospitalisations and 2,255 were deaths. Table 1 shows, for each urban area, the distribution of the census sections, the number of inhabitants, the AGI and confirmed COVID-19 cases (infections, hospitalisations and deaths) during the second time window studied. The confirmed cases of COVID-19 that were declared in the first wave made up only 4.8 per cent of the total number of declared cases during the study period^20^. As mentioned in the methodology, we mainly report findings from the second and third waves (the distribution of confirmed cases during the first wave and their relative contribution to the overall study period is shown in Table 2).

**Table 1:** Population, census sections, income and confirmed COVID-19 cases of hospitalisation and death in the 2nd and 3rd waves, by urban area

**MUNICIPALITY**	**POPULATION 2020 (NSI)**	**Census sections**	**AGI (range)**	**AGI Median (IQR)**	**Cases of COVID-19**	**Cases** /**100.000**	**Hospitalised COVID-19**	**Hospitalised /100.000**	**Deaths COVID-19**	**Deaths /100.000**
**Sevilla**	691,395	531	13,392–123,653	33,612 (22,321)	26,527	3836.74	2334	337.58	418	60.46
**Málaga**	578,490	434	13,392–104,651	29,712 (13,041)	22,848	3949.59	1691	292.31	280	48.4
**Córdoba**	326,039	249	13,392–122,676	30,515 (15,533)	15,095	4629.81	1208	370.51	168	51.53
**Granada**	233,648	184	13,392–68,458	35,232 (17,677)	16,525	7072.6	1422	608.61	304	130.11
**Jerez**	213,105	165	13,615–75,403	26,712 (11,185)	8712	4088.13	618	290	155	72.73
**Almería**	201,322	133	13,392–69,606	31,765 (13,354)	11,890	5905.96	723	359.13	131	65.07
**Marbella**	147,633	77	22,984–52,582	31,812 (7496)	6710	4545.05	397	268.91	88	59.61
**Huelva**	143,837	109	14,934–66,261	30,560 (14,264)	5085	3535.25	363	252.37	56	38.93
**Dos Hermanas**	135,050	83	15,399–94,181	32,416 (14,054)	5258	3893.37	352	260.64	67	49.61
**Algeciras**	123,078	87	14,078–74,990	32,915 (13,386)	4784	3886.97	361	293.31	120	97.5
**Cádiz**	115,439	107	20,961–84,286	33,103 (14,381)	4853	4203.95	385	333.51	52	45.05
**Jaén**	112,757	87	18,463–86,933	35,078 (14,343)	5738	5088.82	412	365.39	70	62.08

**Table 2:** Comparison of total cases, hospitalisations and deaths, by time window and urban area

MUNICIPALITY	1st wave. Cases	2nd-3rd waves. Cases	Totalcases	% Cases 1st wave	1st wave. Hospitalisations (% of cases)	2nd-3rd waves. Hospitalisations (% of cases)	Total hospitalisations	% Hospitalisations 1st wave	1st wave. Deaths (% of cases)	2nd-3rd waves. Deaths (% of cases)	Total deaths	% Deaths 1st wave
Sevilla	988	26,527	27,515	3.59	381 (38.6%)	2334 (8.8%)	2715	14.03	48 (4.9%)	418 (1.6%)	466	10.30
Málaga	1977	22,848	24,825	7.96	644 (32.6%)	1691 (7.4%)	2335	27.58	105 (5.3%)	280 (1.2%)	385	27.27
Córdoba	697	15,095	15,792	4.41	235 (33.7%)	1208 (8.0%)	1443	16.28	21 (3.0%)	168 (1.1%)	189	11.11
Granada	1164	16,525	17,689	6.58	385 (33.1%)	1422 (8.6%)	1807	21.30	67 (5.8%)	304 (1.8%)	371	18.05
Jerez	222	8712	8934	2.48	109 (49.1%)	618 (7.1%)	727	14.99	18 (8.1%)	155 (1.8%)	173	10.40
Almería	211	11,890	12,101	1.74	53 (25.1%)	723 (6.1%)	776	6.82	5 (2.4%)	131 (1.1%)	136	3.67
Marbella	312	6710	7022	4.44	94 (30.1%)	397 (5.9%)	491	19.14	14 (4.5%)	88 (1.3%)	102	13.72
Huelva	175	5085	5260	3.32	70 (40.0%)	363 (7.1%)	433	16.16	14 (8.0%)	56 (1.1%)	70	20
Dos Hermanas	155	5258	5413	2.86	59 (38.1%)	352 (6.7%)	411	14.35	7 (4.5%)	67 (1.3%)	74	9.45
Algeciras	165	4784	4949	3.33	60 (36.4%)	361 (7.5%)	421	14.25	15 (9.1%)	120 (2.5%)	135	11.11
Cádiz	200	4853	5053	3.95	62 (31.0%)	385 (7.9%)	447	13.87	10 (5.0%)	52 (1.1%)	62	16.12
Jaén	452	5738	6190	7.30	167 (36.9%)	412 (7.2%)	579	28.84	22 (4.9%)	70 (1.2%)	92	23.91

### COVID-19 cases

**Fig. 3:**
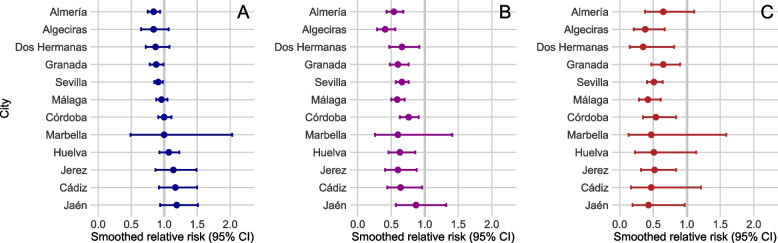
Smoothed relative risks for cases of infection (**a**), hospitalisation (**b**) and death (**c**) due to COVID-19, by household annual gross income, 2nd and 3rd waves

In the second and third waves, the urban areas with the greatest number of confirmed cases of COVID-19 per 100,000 inhabitants were Granada (7072.6) and Almería (5906); Huelva had the lowest number of cases (3535.3). The distribution of the incidence of COVID-19 by urban area is shown in Supplementary File 2. The relationship between AGI and SIR at the case level varied by urban area; however, it only reached statistical significance in the municipalities with a negative association, that is to say, there was greater risk in the census sections with lower income (Fig. 3-A and Supplementary File 3). These included Almería (SRR = 0.84, 95% credibility interval [CI] = 0.75–0.94), Granada (SRR = 0.88, 95%CI = 0.78–1.00) and Sevilla (SRR = 0.91, 95%CI 0.85–0.98).

### Hospitalised cases

In the second and third waves, the number of confirmed cases of COVID-19 that were hospitalised per 100,000 inhabitants was homogeneous among the urban areas, except for Granada (608.6), with an incidence much higher than the next highest (Córdoba, with 370.5). The association between AGI and the SIR of COVID-19 in terms of hospitalisations was negative in all of the municipalities and reached statistical significance in all except Marbella and Jaén (Fig. 3-B and Supplementary File 3). The municipalities in which a lower AGI at a census section level was associated with a greater relative risk of hospitalisation were Algeciras (SRR = 0.41, 95%CI = 0.29–0.56) and Almería (SRR = 0.54, 95%CI = 0.43–0.68).

### Deaths

In the second and third waves, the heterogeneity observed in the number of deaths due to COVID-19 per 100,000 inhabitants was similar to what was described for the first wave. Granada (130.7) was again the municipality with the greatest number of deaths, followed by Algeciras (97.5), while the municipality with the lowest number of deaths was Huelva (38.9). The association between AGI and the SIR of COVID-19 in terms of deaths was negative in all of the urban areas and reached statistical significance in eight of the twelve (Fig. 3-C and Supplementary File 3). The municipalities in which a lower AGI at a census section level was associated with a greater relative risk of death due to COVID-19 were Dos Hermanas (SRR = 0.35, 95%CI = 0.15–0.81), Algeciras (SRR = 0.38, 95%CI = 0.21–0.67), and Málaga (SRR = 0.42, 95%CI = 0.29–0.61).

## Discussion

This study suggests that infections, hospitalisations and deaths related to COVID-19 were greater in underprivileged areas. This confirms what has been shown in prior studies both in Spain [10, 24] and internationally [25, 26], though it provides some important nuances: the present study was carried out in an urban area, but the results are based on 12 urban areas (rather than one) with populations between 100,000 and 600,000 inhabitants. Furthermore, it is a simultaneous study of cases, hospitalisations and deaths. The association between low socioeconomic level and COVID-19 exists, but it also seems that the magnitude tends to increase along with the severity of the disease.

This study was based on the differences within various urban areas in which there were different socioeconomic levels, each one of which had results that were homogeneous, thus showing large income inequalities within each municipality (but not between municipalities). The behaviour of the pandemic throughout the second and third waves was much less geographically heterogeneous than the first [14]. Although this study was of the 12 most populated cities in a Spanish region, it is still a region with 8.5 million people and a territorial extension larger than Ireland or Austria. This meant that in certain cities such as Granada, the number of cases, hospitalisation and deaths due to COVID-19 was practically double that of Sevilla. These differences could be explained by city characteristics, such as population density, environmental and other climatic factors [24, 27, 28]. However, the fact that census sections with lower income were hit hardest was a similar attribute among all of the areas studied. A 10 per cent increase in risk of infection, 40 per cent increase in risk of hospitalisation, and 100 per cent increase in risk of death was observed for each decrease of one logarithmic unit in annual AGI (for example, the risk of death with a COVID-19 diagnosis during the study period in a census section with an average AGI of 15,000 € was double that of a census section with 40,000 €). This inverse relationship between socioeconomic level and the entire cascade from COVID-infection to mortality had been pointed out previously in other high-income countries [25, 29, 30]. However, the present study shows a more pronounced relationship between lower level and greater severity of the disease.

Referring to cases of infection, the areas with lower income were more affected. This has already been shown in terms of the confirmed cases in other Spanish cities such as Madrid [31] and Barcelona [7, 10] and other areas such as New York [32]. Several reasons have been pointed out to explain the greater infection rate among lower income areas: living in smaller houses that do not allow for social distancing [17]; difficulties of telework for less-qualified jobs [33]- and also along these lines, the economic component; having lost a source of income would prompt households to search for other options [7]-; and more challenges with the use of private transportation [34]. It has also been pointed out that the information on prevention measures has been received by the population in part based on their cultural level [35], which, in developed countries tends to be associated with income level.

One of the most novel aspects of this research is the joint study, in a single population, not only of cases but also of hospitalisations and deaths. The explanations described prior for greater contagion of COVID (crowding, work instability, use of transport) are not useful in the latter cases. The greater rates of hospitalisation and death depend on other mechanisms. There are at least two possible explanations related to this relationship.

On the one hand, it could be that these rates were a direct reflection of poorer access to health services of those with low income [36], especially primary care [37]. This could have delayed the early management of symptoms and increased both the possibilities of hospitalisations and of death. The problem of health access can be approached in two ways. One is “physical” in that greater problems contacting and attending health centres could be aggravated by the collateral impact of COVID-19 on the whole system. The COVID-19 crisis has changed the Spanish National Health System [38]. Operations were suspended, visits were delayed and accessing the system became more complicated. Second, there is also the issue of health culture or the health empowerment of citizens. Not everyone knows how to correctly interpret the signs of severity that should prompt them to seek health services nor how to provide informal home care. If to this we add the lack of knowledge of the disease, this could have been a notable barrier. During the crisis, it became clear that education should be improved in this sense.

On the other hand, the greater impact on those with lower income could be explained, in addition to worse health access, by the life circumstances of these people. A lower socioeconomic level is related to worse health [39]. Also, pre-existing conditions prior to contracting COVID-19 have been shown to be related to a more severe development of the disease [40].

Thus, if the characteristics of the environment are relevant in contracting COVID, the access to health services and prior levels of health determine the level of affectation. This could explain why inequalities have been especially evident in hospitalisations and deaths. We also cannot rule out that this finding could go beyond the illnesses related to COVID-19. Between the beginning of the pandemic and July of 2021, Spain had excess mortality of over 85,000 deaths [15]. We do not know how many are related to the collapse of the health system or whether this could have a greater impact on lower socioeconomic levels, but results such as these should inspire further investigation in this sense. What seems clear, according to this study, is that the pandemic has exacerbated existing structural inequities, though not in a uniform way, over the past year and a half.

In terms of study limitations, this is an ecological study, and the mechanisms underlying the associations between living in poor areas and incidence, hospitalisation and mortality during the COVID-19 pandemic can only be hypothesised [22]. Secondly, this study excluded institutionalised people. The reason for this was that it was impossible to link these individuals with their socioeconomic level based on their households, because in most cases the geocoding corresponded to the centre where they were institutionalised. As a result, the risk of information bias was reduced but this may have also caused a selection bias. Furthermore, the data related to the first wave were excluded from the analysis, which reduced the number of cases to study. However, this constitutes more a strength than a limitation, because given the biases in reporting of COVID-19 cases during the first wave- mentioned earlier- these data are unreliable and could compromise the internal validity of the study. Thirdly, the AGI was taken from the most recent year available (2018), so we might have overestimated its values; this all the more applies to the census sections in which the AGI between 2015 and 2017 was used (0.7%). However, compared to studies from other European countries, this potential bias might be mitigated by the social measures implemented by the Spanish government in 2020, aimed at protecting individuals who became unemployed or partially employed. Fourthly, our results may be only generalisable to large urban areas from high-income countries which adopted non-pharmaceutical interventions similar to those adopted in Spain. Finally, although the incidence rates were standardised by sex and age, the proportionality between the specific rates per subgroup in each census section with respect to the rest could not be verified, which may have led to a reduction in the accuracy of the relative risk [41]. Thus, the point estimates yielded by the statistical models should be interpreted with caution.

This study also has strengths. The most important is that in a single study, low socioeconomic status was linked not only to COVID-infection, but also to hospitalisations and deaths. This had not been done in Spain until now. Moreover, our study used more than one urban area and smaller geographical scale than previous studies [22, 31].

## Conclusions

Our results indicate the existence of income inequalities by geographical area in the incidence of cases, hospitalisations and deaths due to COVID-19, adjusted by age and sex. They suggest that a lower socioeconomic level is related not only to a greater risk of infection by COVID-19, but also greater related complications.

Selective actions are needed to improve the population´s capacity to protect itself from infection and obtain conditions of equal access to health services. Such a need is not exclusive to COVID-19, as these results show inequalities that existed prior to the onset of the pandemic, which -if unaddressed- might persist and even become worse in the future.

## Supplementary Information

**Additional file 1:** **Supplementary File 1.** Distribution of annual average gross income per household, by urban area. **Supplementary File 2.** Distribution of the smoothed standardised incidence ratio (SIR) of infection in the second and third epidemic waves, by urban area. **Supplementary File 3.** Results of the spatial regression model, by time window and urban area.

## Data Availability

Data will be made available upon reasonable request by contacting the corresponding author. Andalusia COVID-19 data are published up to the municipal level on the website (https://www.juntadeandalucia.es/institutodeestadisticaycartografia/salud/datosSanitarios.html). Data at census section level, due to its smaller reference population, is not delivered in this format.
