# Supplementary material for: Socioeconomic differences in COVID-19 infection, hospitalisation and mortality in urban areas in a region in the South of Europe
Source: BMC Public Health. 2022 Dec 12;22:2316. doi: 10.1186/s12889-022-14774-6 (PMC9742010; doi:10.1186/s12889-022-14774-6)
Supplement: Supplementary file 1 — Additional file 1: Supplementary File 1. Distribution of annual average gross income per household, by urban area. Supplementary File 2. Distribution of the smoothed standardised incidence ratio (SIR) of infection in the second and third epidemic waves, by urban area. Supplementary File 3. Results of the spatial regression model, by time window and urban area. [file 12889_2022_14774_MOESM1_ESM.docx]

**Supplementary File 1. Distribution of annual average gross income per household, by urban area.**


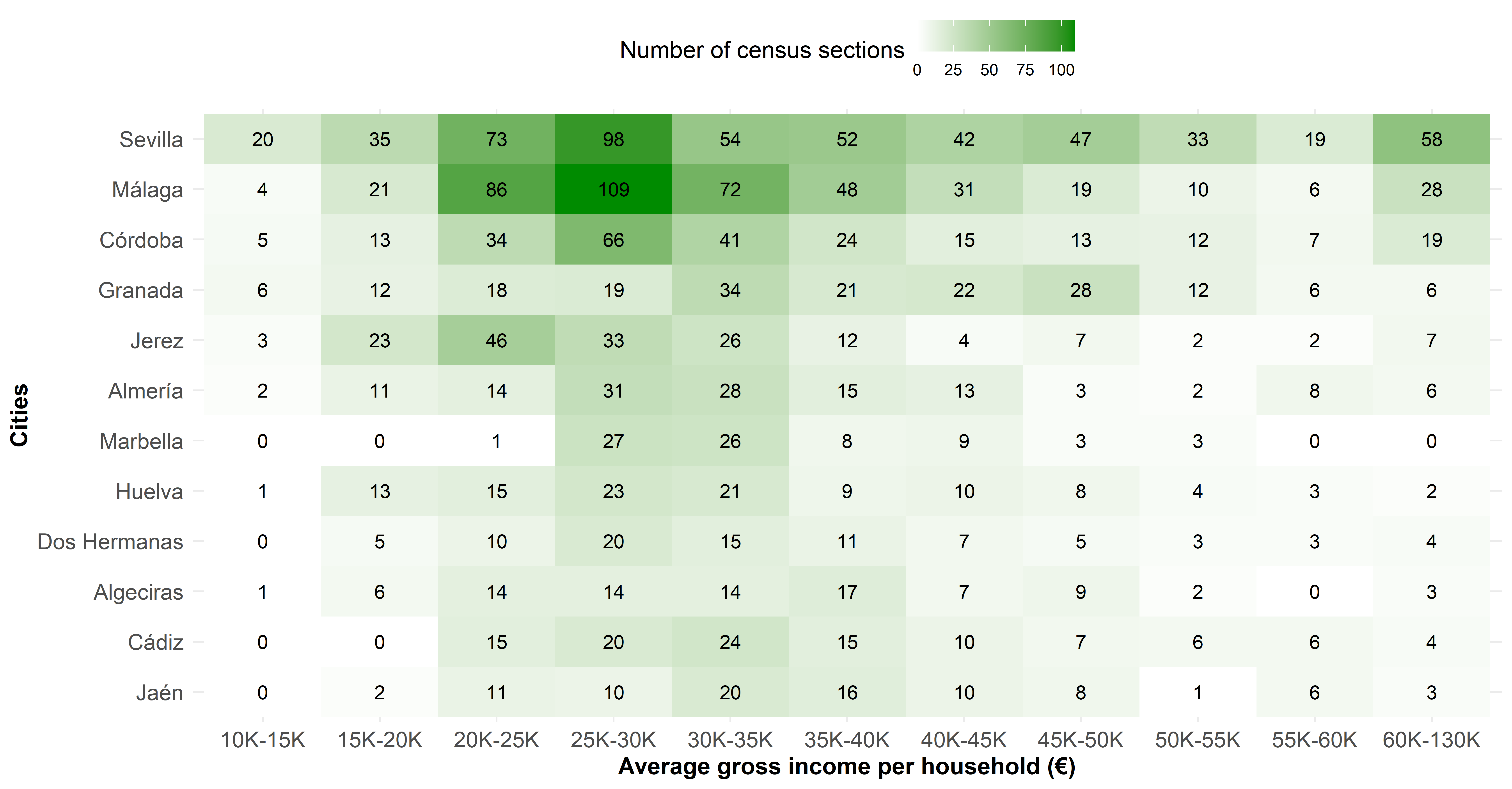


*Average gross income's last category represents a substantially larger income range.

**Supplementary File 2. Distribution of the smoothed standardised incidence ratio (SIR) of infection in the second and third epidemic waves, by urban area.**


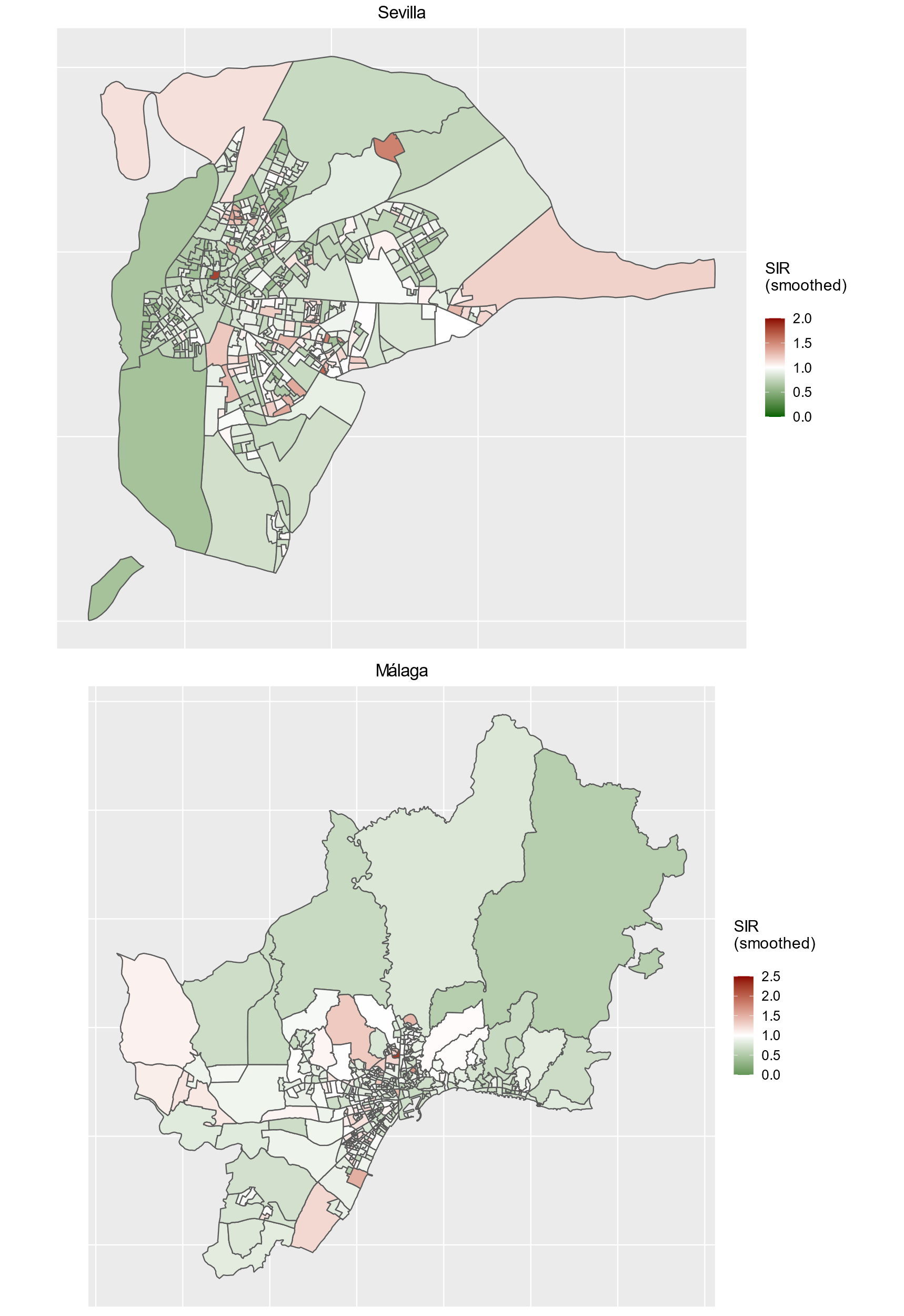


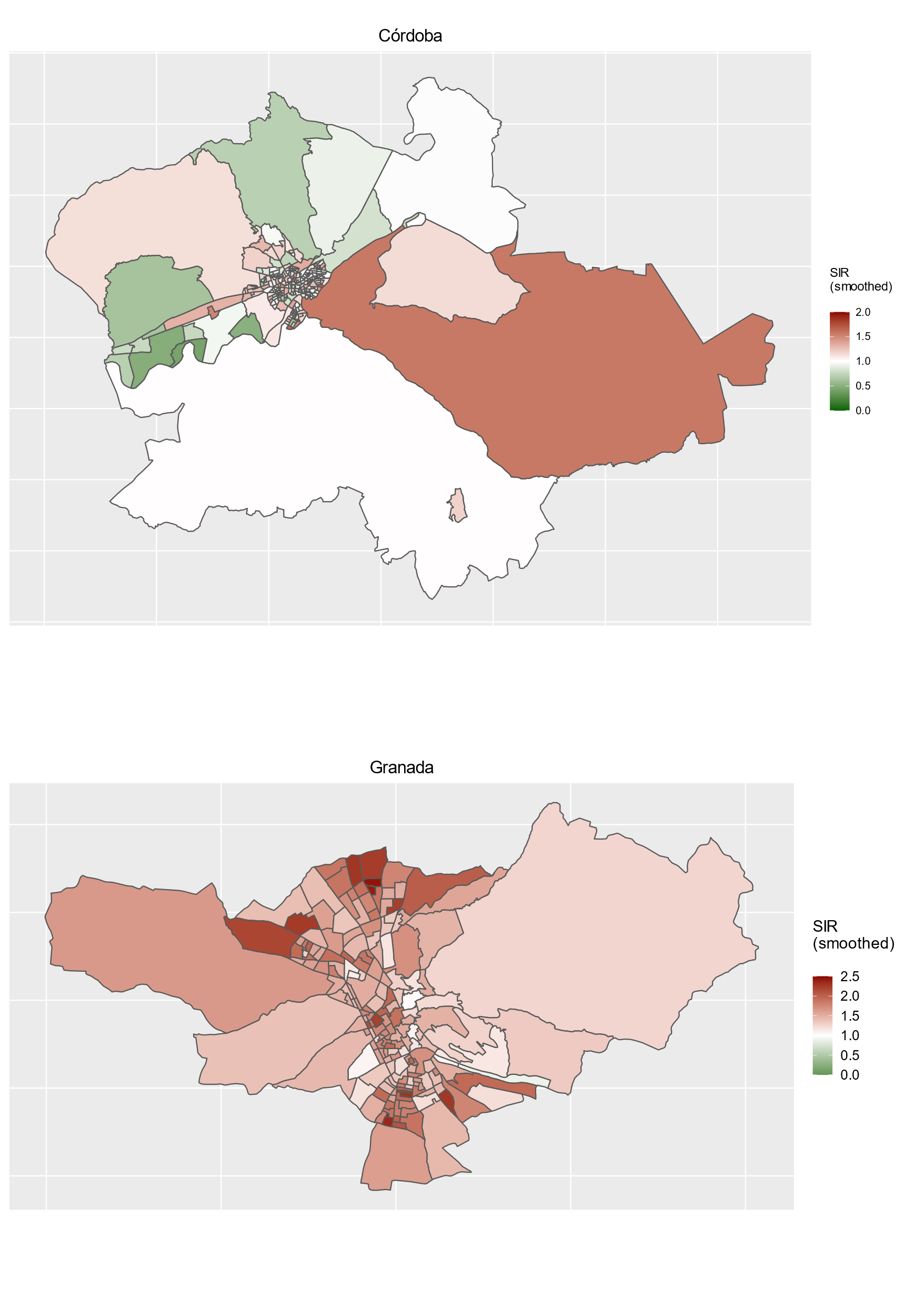


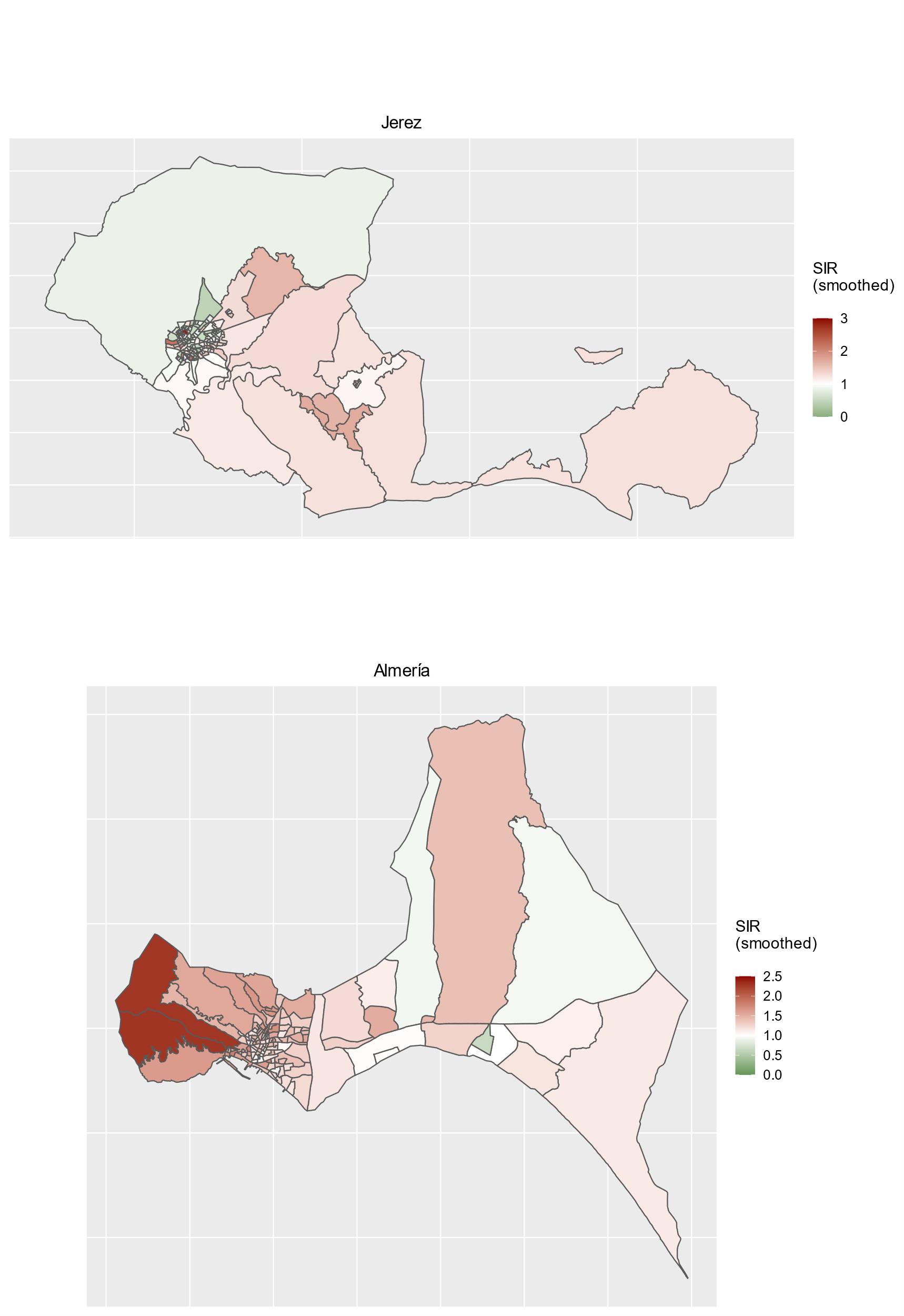


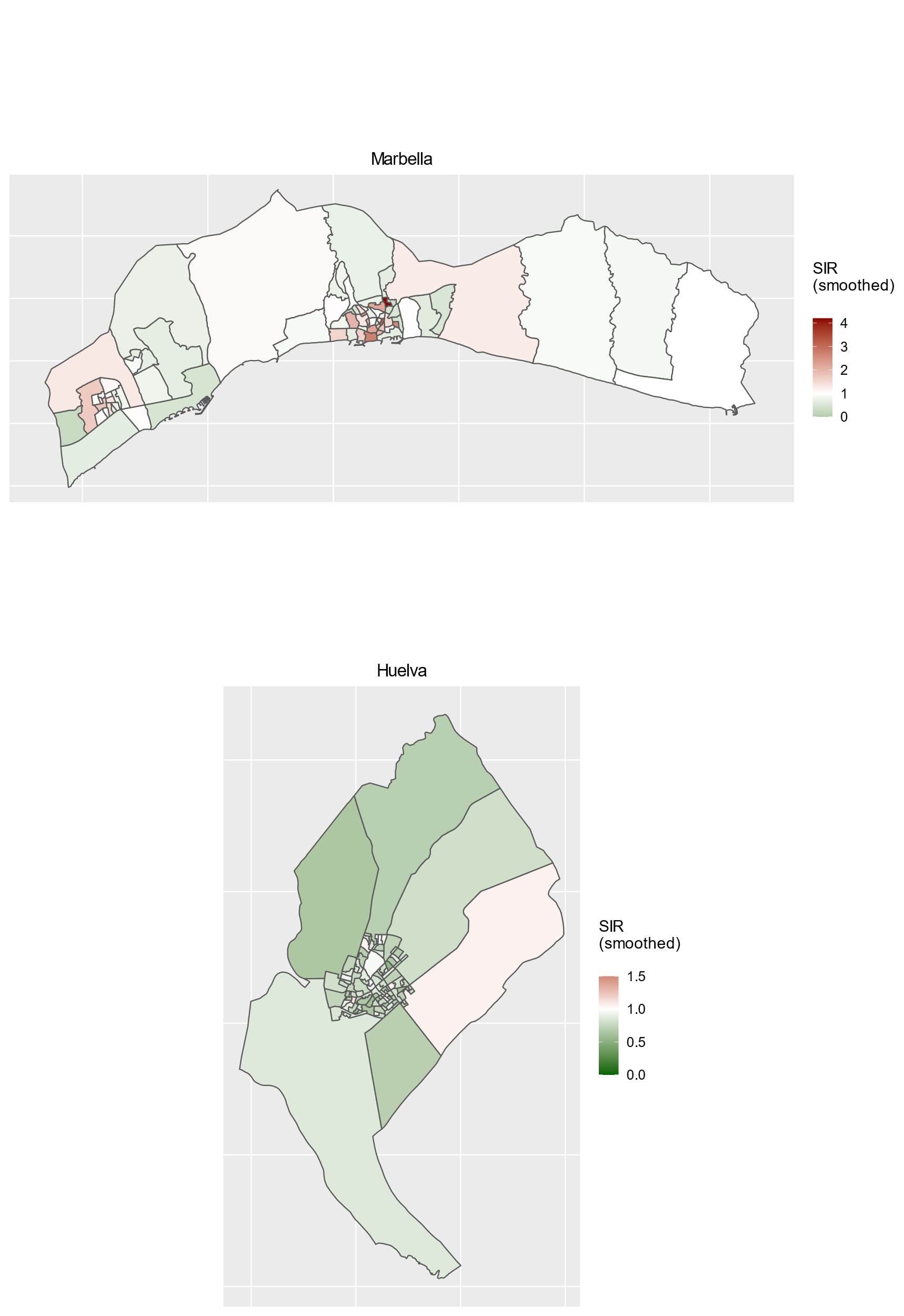


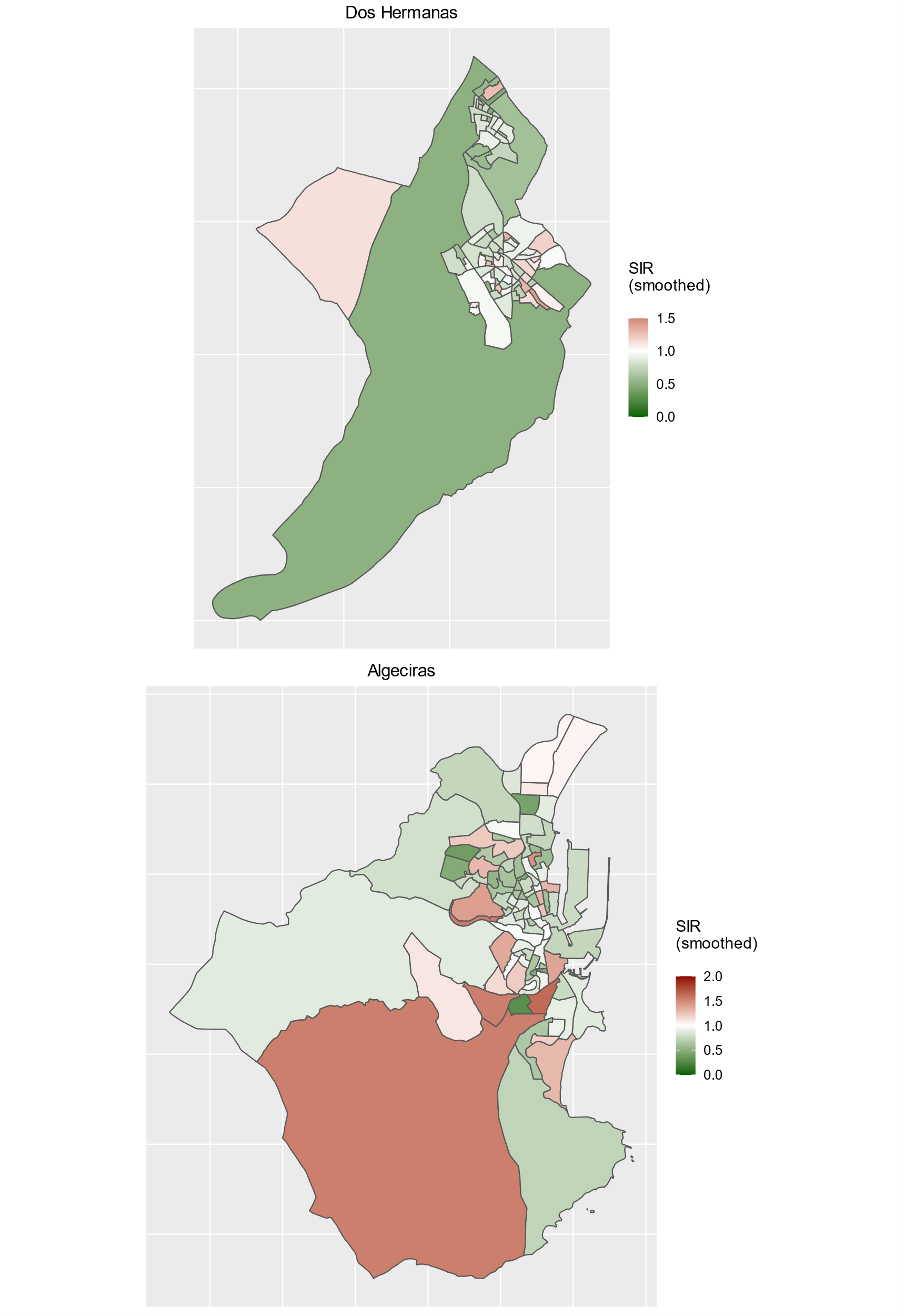


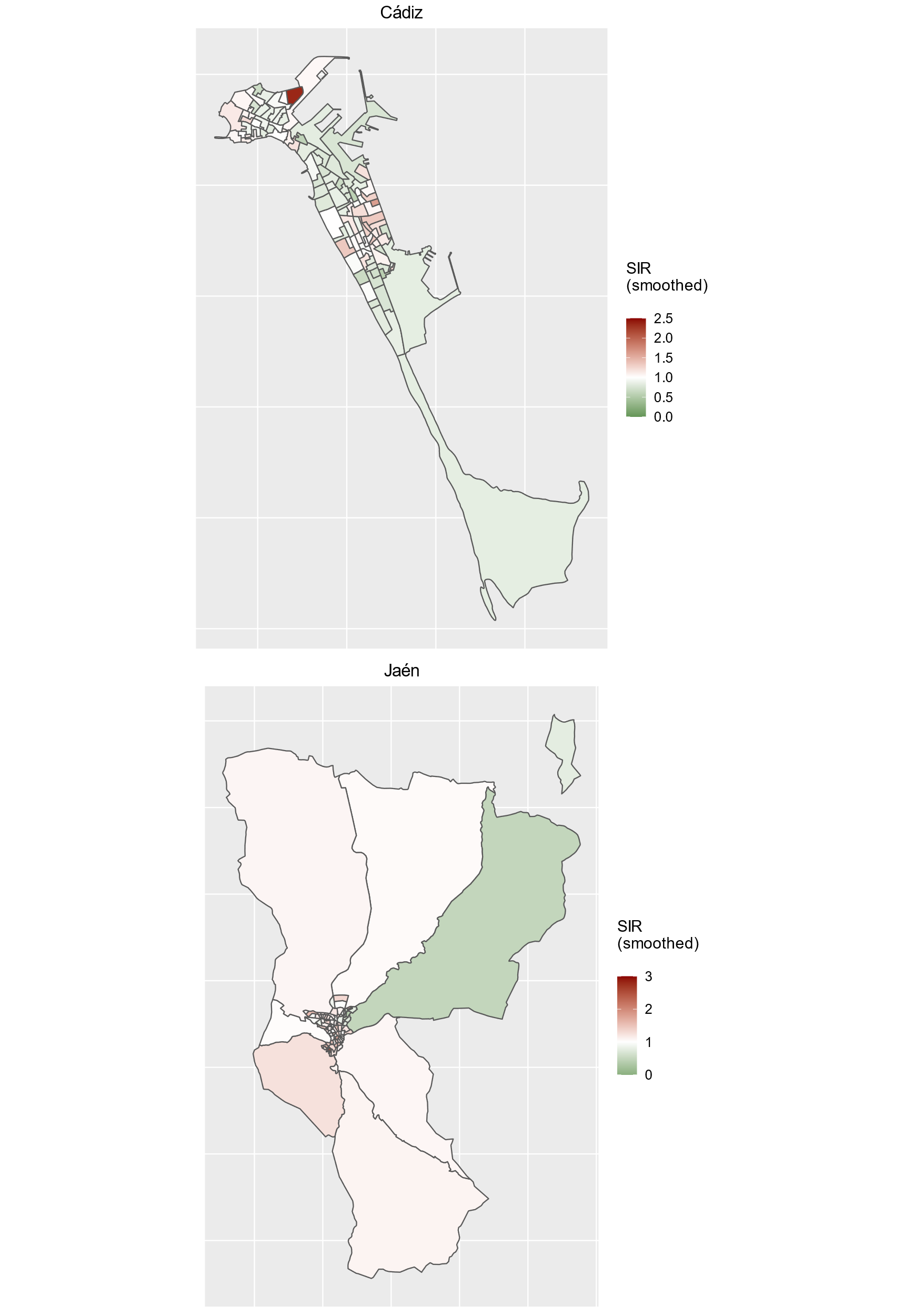


**Supplementary File 3. Results of the spatial regression model, by time window and urban area.**

| **MUNICIPALITY** | **RR 1st wave.  Cases (95% CI)** | **RR 2nd-3rd wave Cases (95% CI)** | **RR 1st wave. Hospitalizations (95% CI)** | **RR 2nd-3rd wave. Hospitalizations (95% CI)** | **RR 1st wave.  Deaths (95% CI)** | **RR 2nd-3rd wave. Deaths (95% CI)** |
| --- | --- | --- | --- | --- | --- | --- |
| Sevilla | **2.40 (2.01-2.87)*** | **0.91 (0.85-0.98)*** | **1.51 (1.12-2.10)*** | **0.66 (0.57-0.76)*** | 0.97 (0.50-1.85) | **0.51 (0.41-0.64)*** |
| Málaga | **2.51 (2.09-3.02)*** | 0.96 (0.88-1.05) | **1.54 (1.18-2.06)*** | **0.59 (0.50-0.70)*** | 0.87 (0.49-1.49) | **0.42 (0.29-0.61)*** |
| Córdoba | **2.32 (1.84-2.94)*** | 1.00 (0.91-1.11) | **1.47 (1.05-2.04)*** | **0.76 (0.63-0.91)*** | 0.67 (0.19-2.18) | **0.54 (0.35-0.84)*** |
| Granada | **1.66 (1.29-2.16)*** | **0.88 (0.78-1.00)*** | 1.04 (0.78-1.39) | **0.60 (0.48-0.76)*** | 1.01 (0.51-2.07) | **0.65 (0.47-0.90)*** |
| Jerez | 1.28 (0.88-1.85) | 1.14 (0.87-1.49) | 0.98 (0.57-1.66) | **0.60 (0.41-0.88)*** | 0.48 (0.11-1.87) | **0.52 (0.32-0.84)*** |
| Almería | **3.94 (2.62-5.90)** | **0.84 (0.75-0.94)*** | **3.61 (1.62-8.08)*** | **0.54 (0.43-0.68)*** | 0.23 (0.01-3.82) | 0.65 (0.38-1.11) |
| Marbella | **2.09 (1.09-3.93)*** | 1.00 (0.49-2.03) | **4.12 (1.41-11.75)*** | 0.60 (0.26-1.41) | 2.91 (0.15-45.38) | 0.47 (0.13-1.59) |
| Huelva | **1.70 (1.09-.69)*** | 1.07 (0.93-1.23) | 0.77 (0.38-1.56) | **0.63 (0.46-0.86)*** | **0.18 (0.03-0.89)*** | 0.51 (0.23-1.14) |
| Dos Hermanas | **2.83 (1.79-4.47)*** | 0.87 (0.72-1.08) | **2.35 (1.12-4.84)*** | **0.66 (0.47-0.92)*** | 3.85 (0.42-28.42) | **0.35 (0.15-0.81)*** |
| Algeciras | 1.28 (0.80-2.04) | 0.84 (0.65-1.07) | **0.42 (0.19-0.91)*** | **0.41 (0.29-0.56)*** | **0.18 (0.03-0.90)*** | **0.38 (0.21-0.67)*** |
| Cádiz | **3.46 (2.01-5.91)*** | 1.17 (0.92-1.50) | **3.48 (1.53-7.84)*** | **0.64 (0.44-0.96)*** | 0.65 (0.07-5.19) | 0.47 (0.17-1.21) |
| Jaén | **3.36 (2.32-4.88)*** | 1.19 (0.94-1.51) | **2.60 (1.55-4.35)*** | 0.87 (0.57-1.32) | 3.06 (0.72-13.07) | **0.43 (0.19-0.97)*** |

RR: Relative risk (smoothed); CI: credibility interval. Asterisks indicate statistically-significant associations.
